# Polarization-dependent phase-modulation metasurface for vortex beam (de)multiplexing

**DOI:** 10.1515/nanoph-2022-0710

**Published:** 2023-02-20

**Authors:** Haisheng Wu, Qingji Zeng, Xinrou Wang, Canming Li, Zebin Huang, Zhiqiang Xie, Yanliang He, Junmin Liu, Huapeng Ye, Yu Chen, Ying Li, Dianyuan Fan, Shuqing Chen

**Affiliations:** International Collaborative Laboratory of 2D Materials for Optoelectronics Science and Technology, Institute of Microscale Optoelectronics, Shenzhen University, Shenzhen 518060, China; College of New Materials and New Energies, Shenzhen Technology University, Shenzhen 518118, China; South China Academy of Advanced Optoelectronics, Guangdong Provincial Key Laboratory of Optical Information Materials and Technology and Institute of Electronic Paper Displays, South China Normal University, Guangzhou 510006, China; International Collaborative Laboratory of 2D Materials for Optoelectronics Science and Technology, Institute of Microscale, Shenzhen, China

**Keywords:** metasurface, orbital angular momentum mode, polarization-dependent phase modulation, polarization-division-multiplexing

## Abstract

Vortex beams (VBs) carrying orbital angular momentum (OAM) have shown promising potential in enhancing communication capacity through the possession of multiple multiplexing dimensions involving the OAM mode, polarization, and wavelength. Although many research works on multidimensional multiplexing have been conducted, the (de)multiplexer compatible with these dimensions remains elusive. Following the expanded concept of the Pancharatnam–Berry (PB) phase, we designed a polarization-dependent phase-modulation metasurface to phase-modulate the two orthogonal linearly polarized components of light, and two Dammann vortex gratings with orthogonal polarization responses were loaded to simultaneously (de)multiplex OAM mode and polarization channels. As a proof of concept, we constructed a 16-channel multidimensional multiplexing communication system (including two OAM modes, two polarization states, and four wavelengths), and 400 Gbit/s quadrature-phase shift-keying (QPSK) signals were transmitted. The results demonstrate that the OAM mode and polarization channels are successfully (de)multiplexed, and the bit-error-rates (BERs) are below 1.67 × 10^−6^ at the received power of −15 dBm.

## 1 Introduction

Vortex beams (VBs) possessing orbital angular momentum (OAM) [1–4] are emerging as a powerful carrier for ultrahigh-speed optical communication owing to their mode orthogonality [5–7]. Besides, OAM mode is independent of traditional physical dimensions involving wavelength and polarization, indicating that it can combine with polarization-division-multiplexing (PDM) and wavelength-division-multiplexing (WDM) [8–12] to further enlarge communication capacity. By multiplexing the OAM mode, polarization, and wavelength, the communication capacity has improved to Pbit/s [13, 14]; however, these channels are often performed individually owing to the lack of multi-dimensional (de)multiplexers, which results in severe energy loss and system redundancy. In addition, offline equalization algorithms are always inevitable in processing PDM signals, further restricting their application to all-optical interconnections and networks. Limited by the exhaustion of spectral resources, it is difficult to increase communication capacity simply by increasing the multiplexed wavelength channels. Hence, a (de)multiplexer that can simultaneously (de)multiplex OAM mode and polarization channels and is compatible with WDM is urgently needed for the practical application of VB multiplexing communication.

Numerous phase-modulation devices have been proposed for the (de)multiplexing of OAM mode channels, including spiral phase plates [15], Q-plates [16], and spatial light modulators (SLM) [17, 18]. Spiral phase plates and Q-plates with azimuthally rotated phase differences are used to generate OAM modes, but they can only handle a single OAM mode. SLM can load different holograph grating phases to (de)multiplex multi-OAM modes, but the (de)multiplexing of polarization dimension remains a significant challenge because liquid crystal devices lack phase modulation capability with polarization selectivity. Metasurface [19–25], an artificial microstructure array, has shown independent control capacity for multiple physical dimensions by properly designing the sizes and azimuthal angles of meta-atoms. A common scheme is loading *l*-fork grating phases in Pancharatnam–Berry (PB) phase metasurface to multiplex OAM modes, but the lack of polarization-dependent phase modulation capacity limits its ability to multiplex polarization channels. Utilizing the polarization selectivity of propagation phase metasurfaces, researchers have coupled/separated OAM mode and polarization channels [26–28]; however, the size-dependent anisotropy meta-atom design results in wavelength sensitivity, which means it cannot be compatible with WDM. Hence, the (de)multiplexer for OAM mode and polarization channels with a broad working wavelength remains a challenge in high-speed optical communication.

Herein, a polarization-dependent phase-modulation metasurface is proposed and experimentally demonstrated for the simultaneous (de)multiplexing of the OAM mode and polarization channels. By exploiting the expanded concept of the PB phase, a rectangular meta-atom is introduced to phase-modulate the two orthogonal linearly polarized components of light by rotating the azimuthal angle, and two Dammann vortex grating phases with orthogonal polarization responses are imposed on the metasurface to (de)multiplex the OAM mode and polarization channels. By virtue of the dispersion-free characteristic of the PB phase, this metasurface has a broad working wavelength ranging from 1535 nm to 1607 nm to be compatible with WDM. As a proof of concept, we constructed a 16-channel VB multiplexing communication system (including two OAM modes, two polarization states, and four wavelengths), and 400 Gbit/s quadrature-phase shift-keying (QPSK) signals were successfully transmitted. The results show that the OAM mode and polarization channels are successfully (de)multiplexed with bit-error-rates (BERs) of 1.67 × 10^−6^ at a received optical power of −15 dBm. Due to the flexible design of Dammann vortex grating, this metasurface can further multiplex more OAM modes to further enlarge communication capacity. This technique paves the way for the integration of multidimensional multiplexing communication systems [29, 30] and large-scale dense data communication [31, 32].

## 2 Principles

**Figure 1:**
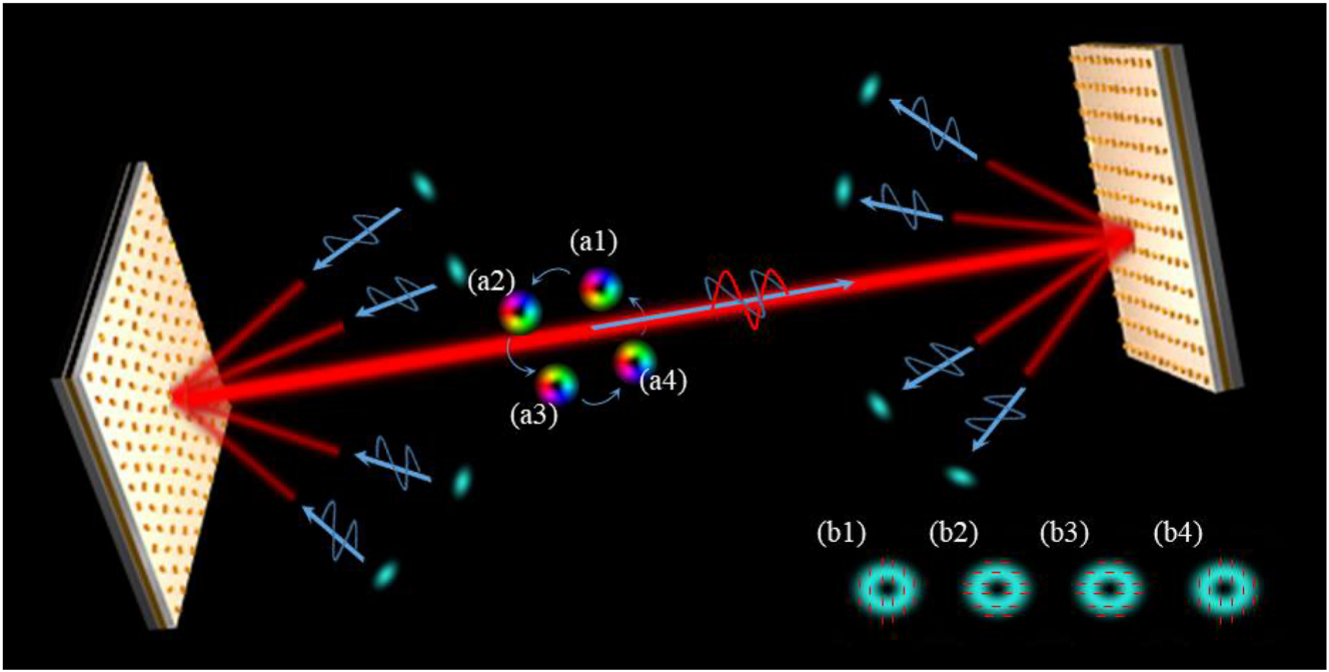
The mechanism of (de)multiplexing with polarization and OAM mode. *X*-polarized Gaussian beams incident on the multiplexer at different diffraction angles. Then the VBs with different OAM modes and *x*- or *y*-polarization states can be generated and coaxially transmit along with the zeroth diffraction order. At receiver, the OAM mode and polarization channels are reconverted into Gaussian beams and separated spatially for detection by the demultiplexer. (a1)–(a4) The complex amplitude distribution of four reflected beams. (b1)–(b4) The polarization distribution of four reflected beams.

A conceptual diagram depicting the mechanism of (de)multiplexing the OAM mode and polarization channels is shown in Figure 1. Four *x*-polarized Gaussian beams carrying optical signals are incident on the metasurface at angles of different diffraction orders, and the VBs with different OAM modes and *x*- or *y*-polarization state can be generated and coaxially transmit along with the zeroth diffraction order. At the receiver, another identical metasurface is used to demultiplex all channels according to the OAM mode and polarization state. According to the principle of optical path reversibility, these coaxial VBs will be converted into Gaussian mode with *x*-polarization state in their corresponding diffraction orders. Thus, we can simultaneously (de)multiplex the OAM mode and polarization channels.

**Figure 2:**
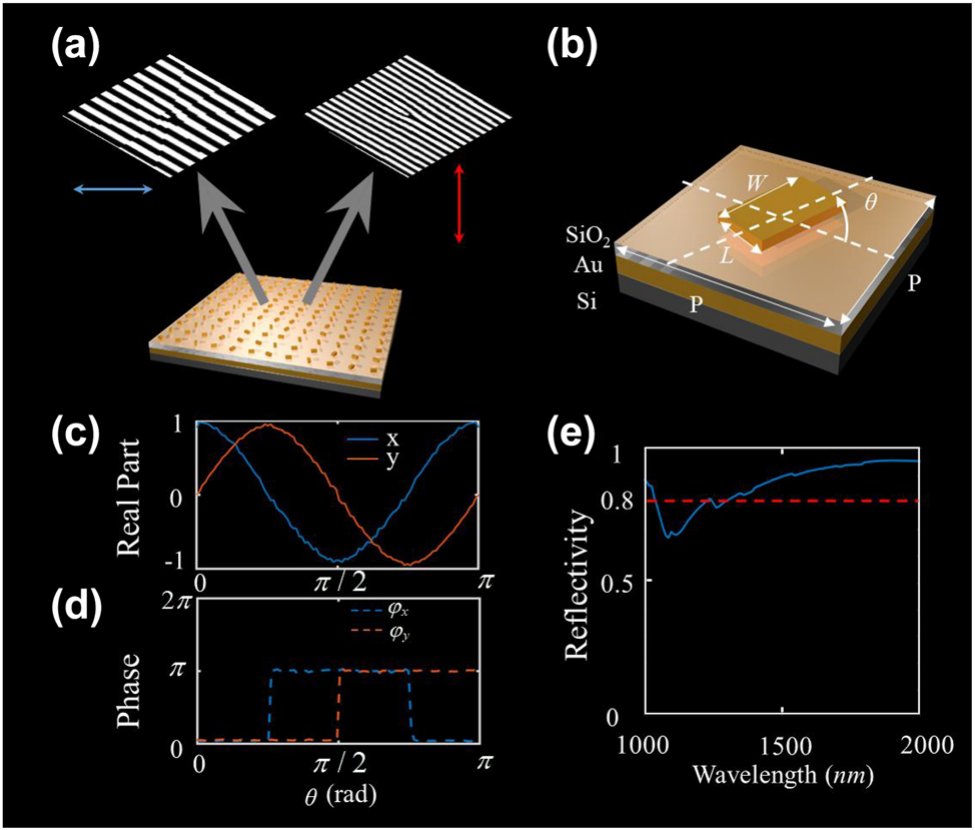
Design of the metasurface-based (de)multiplexer. (a) Two Dammann vortex grating phases with different periods are integrated on the same metasurface with *x*- and *y*-polarization. (b) Geometric parameters of a metaatom. (c) and (d) The real part and phase of *x*- and *y*-polarized components of output field for different orientation angles *θ*. (e) The reflection efficiency from 1000 nm to 2000 nm.

The underlying principle of simultaneously (de)multiplexing the OAM mode and polarization channels is that two different grating phases corresponding to the *x*- and *y*-polarized component can be loaded in the (de)multiplexer. Here, the binarized Dammann vortex grating phase (0 and *π*) is adopted, which can generate different OAM modes at different diffraction orders and provide uniform energy distribution at each diffraction order [33, 34]. As shown in Figure 2(a), the phases of two orthogonal polarized components can be expressed as:

(1) $$\phi_{x,y}=\arg\left(\overset{m=\frac{N}{2}}{\underset{m=-\frac{N}{2}}{\sum }}C_{m}\exp\left[im\left(\frac{2\pi s}{T_{x,y}}+▵l\varphi \right)\right]\right),$$

where *N* is the number of diffraction order, *m* is the diffraction order from−*N*/2 to *N*/2, *C*_*m*_ = 1/*N* is the normalized power of the *m*th diffraction order, *s* represents the phase-turning point in the normalized period, *T*_*x*,*y*_ is the period of the Dammann grating, △*l* is the interval of the topological charges, and *φ* is the azimuthal angle. It is worth mentioning that the diffraction angle of Dammann grating can be adjusted by *T*_*x*,*y*_. To spatially separate the polarization channels to realize polarization (de)multiplexing, the periods of gratings are set as *T*_*x*_ ≠ *T*_*y*_. The superposition field of two Dammann vortex grating with orthogonal polarization responses can be written as:

(2) $$\begin{array}{ll} E_{1} & =\frac{\sqrt{2}}{2}\exp\left(i\phi_{x}\right)\left[\begin{array}{c} 1\\ 0 \end{array}\right]+\frac{\sqrt{2}}{2}\exp\left(i\phi_{y}\right)\left[\begin{array}{c} 0\\ 1 \end{array}\right]\\ & =\frac{\sqrt{2}}{2}\left[\begin{array}{c} \exp\left(i\phi_{x}\right)\\ \exp\left(i\phi_{y}\right) \end{array}\right]. \end{array}$$

Due to *ϕ*_*x*_ and *ϕ*_*y*_ are binarized phase, *E*_1_is discretized into four values ( $\sqrt{2}/2*{\left[1,1\right]}^{T}$ , $\sqrt{2}/2*{\left[1,-1\right]}^{T}$ , $\sqrt{2}/2*{\left[-1,1\right]}^{T}$ , $\sqrt{2}/2*{\left[-1,-1\right]}^{T}$ ). According to Eq. (2), the metasurface-based (de)multiplexer should possess polarization-dependent phase modulation capacity to phase-modulate the two orthogonal linearly polarized components of light and load two Dammann vortex grating phases with orthogonal polarization responses.

The PB phase is one of the most useful schemes for imposing a phase profile by spin-rotation coupling [35–37]. However, the traditional PB phase metasurfaces are phase-only modulation element and perform the transformation of $\left|L\right\rangle =e^{+i2\theta }\left|R\right\rangle$ and $\left|R\right\rangle =e^{-i2\theta }\left|L\right\rangle$ , where *θ* is the orientation angle of meta-atom. In this study, we expanded the concept of the PB phase to phase-modulate the two orthogonal linearly polarized components of light. *X*-polarized light is set as the incident light, and then the outgoing field are related with by the orientation angle of the metaatoms, which can be expressed as follows:

(3) $$\begin{array}{ll} E_{2} & =R(-\theta )\left[\begin{array}{cc} 1 & 0\\ 0 & e^{i\pi } \end{array}\right]R(\theta )\left|x\right\rangle \\ & =\left[\begin{array}{c} \cos\left(2\theta \right)\\ \sin\left(2\theta \right) \end{array}\right]. \end{array}$$

According to Eq. (3), the phase of two orthogonal linearly polarized components (arg(cos(2*θ*)) and arg(sin(2*θ*))) are binarized to 0 and *π*. The amplitudes of two orthogonal linearly polarized components flaw as the functions of2*θ*, but Eq. (2) requires them be equal. However, there are still four values can satisfy this condition ( $\pi /8:\sqrt{2}/2*{\left[1,1\right]}^{T}$ , $7*\pi /8:\sqrt{2}/2*{\left[1,-1\right]}^{T}$ , $3*\pi /8:\sqrt{2}/2*{\left[-1,1\right]}^{T}$ , $5*\pi /8:\sqrt{2}/2*{\left[-1,-1\right]}^{T}$ ), and these values can constitute the complete solution of Eq. (2). By simultaneously solving Eqs. (2) and (3), the unique value of *θ* can be calculated. Hence, the two Dammann vortex grating phases (*ϕ*_*x*_ and *ϕ*_*y*_) with orthogonal polarization responses and periods can be performed by properly designing the optical axis of metasurface.

Based on the theoretical analysis, a metasurface with metal–insulator–metal configuration is designed (see the Supplementary material for detail). Figure 2(b) depicts the basic unit cell, which consists of a gold (Au) meta-atom and a continuous 200 nm-thick Au reflector separated by a 150 nm-thick SiO_2_ dielectric spacer. This multilayer structure can form a Fabry–Perot resonator with low quality factor [38] and the rectangular cross-section leads to different propagation phase along *x*- and *y*-directions (*φ*_*x*_ and *φ*_*y*_). Silicon (Si) is selected as the substrate, and the lattice constant *P* is set to 800 nm to reduce high-order diffraction. The height of the top meta-atom is set to 50 nm to provide sufficient reflection efficiency and phase shift. Considering the half-wave phase delay (*φ*_*x*_ − *φ*_*y*_ = *π*), the length (*L* = 380 nm) and width (*W* = 220 nm) of meta-atom is carefully designed.

The performance of our actual meta-atom is characterized by a finite-difference time-domain (FDTD, Lumerical Solutions) based on the finite element method. Figure 2(c) and (d) shows the *x*- and *y*-polarized components of output field as a function of the orientation angles *θ*. As expected, the real part of *x*- and *y*-polarized components are correspondent to cos(2*θ*) and sin(2*θ*). The phase is binarized to 0 and *π*, which can be described as arg(cos(2*θ*)) and arg(sin(2*θ*)). Thus, the phase of *x*- and *y*-polarized components can be modulated by properly designing the orientation angles *θ*. Finally, owing to the PB phase metasurface generally has a broadband operating wavelength, we further demonstrate the reflectivity in the infrared waveband (see Figure 2(e)). The reflectivity is higher than 80% within the wavelength of 1400 nm–1600 nm.

## 3 Results and analysis

**Figure 3:**
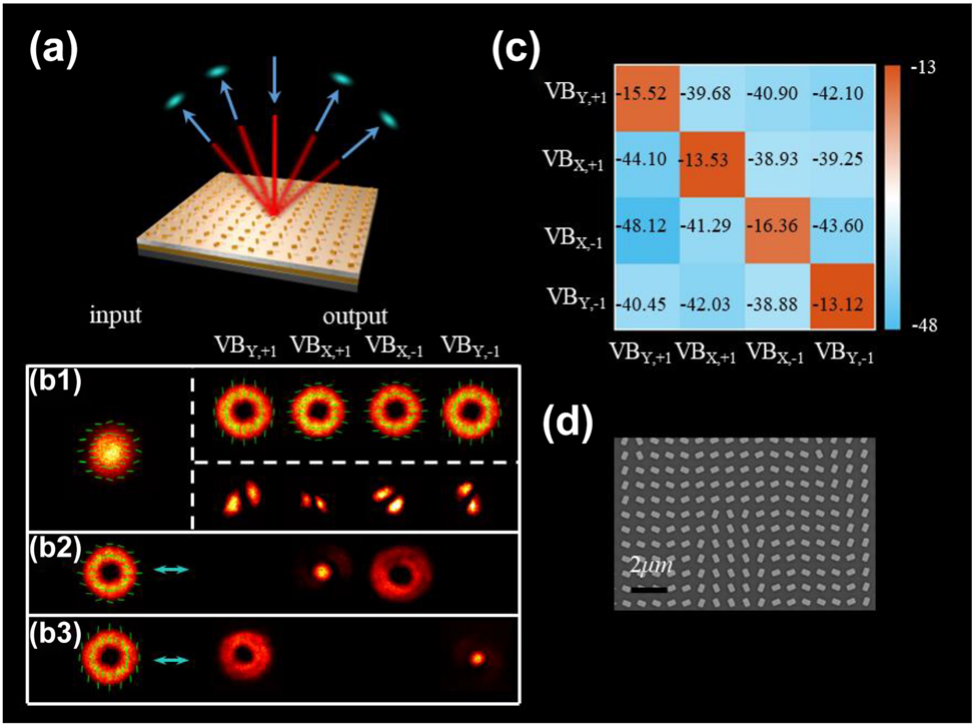
Optical performance of the metasurface-based (de)multiplexer. (a) Optical performance testing system diagram. (b1)–(b3) Polarization and intensity distributions in each diffraction order under different incident beams. (b1) Gaussian beam (*x*-polarization); (b2) VB with *l* = 1 (*x*-polarization); (b3) VB with *l* = −1 (*y*-polarization). (c) The light intensity at the center of each diffraction order under different OAM beams. (d) FE-SEM images of the fabricated (de)multiplexer. The scale of the picture is 2 μm.

For experimental verification, the proposed metasurface was fabricated using the standard electron beam lithography process (see the Supplementary material for fabrication details), and the optical performance of the metasurface was experimentally investigated. As shown in Figure 3(a), an experimental scheme was employed with a light beam normally incident on the metasurface, and the reflected optical field was detected and analyzed. To demonstrate the multiplexing performance of the proposed metasurface, Gaussian beam with *x*-polarization was normally incident onto the metasurface, and then four VBs with different topological charges and polarization states (VB_*Y*,+1_, VB_*X*,+1_, VB_*X*,−1_ and VB_*Y*,−1_, “VB_*Y*,+1_” represents the VB with *l* = 1 and *y*-polarization state) were reflected from the metasurface at different diffraction orders. The topological charges were further measured by the diffraction pattern of cylindrical lens (C-lens). The number of dark stripes represents the modulus of the topological charge and the oblique direction of stripes indicates the charge sign. The measured intensity and polarization distribution of each diffraction order and C-lens diffraction patterns are shown in Figure 3(b1). The energy was uniformly distributed on four diffraction order and the total diffraction efficiency was about 85% In practical multiplexing process, according to the principle of optical path reversibility, four Gaussian beams obliquely incident the metasurface along different diffraction angles, and the VBs with different OAM modes and polarization states can be generated and coaxially emitted along with the zeroth diffraction order.

Then the (de)multiplexing performance of the proposed metasurface was tested. VBs with different topological charges and polarization states (VB_*X*,+1_ and VB_*Y*,−1_) were normally incident on the metasurface, and an additional linear polarizer was inserted to filter the *y*-polarized light. As shown in Figure 3(b2) and (b3), the VBs were completely converted into a Gaussian beam with *x*-polarization at the corresponding diffraction order. To further reveal the detailed crosstalk of this (de)multiplexer, a confusion matrix of four channels was measured. The energy located on the diagonal line in the matrix represents the signal energy of the demultiplexing channel, while the energy of other channel is crosstalk that introduces noise. The crosstalk relates to the polarization orthogonality and mode orthogonality between adjacent channels. As show in Figure 3(c), the energy of the crosstalk is much lower than that of the signal, which demonstrate that the polarization and OAM modes generated by the proposed metasurface have well orthogonality and the proposed (de)multiplexer possesses good communication performance. In addition, the metasurface-based (de)multiplexer has a broadband working wavelength ranging from 1535 nm to 1607 nm owing to the dispersion-free characteristic of the PB phase (see the Supplementary material) [39, 40]. Figure 3(d) depicts the field emission scanning electron microscopy (FE-SEM) images of the fabricated metasurface.

**Figure 4:**
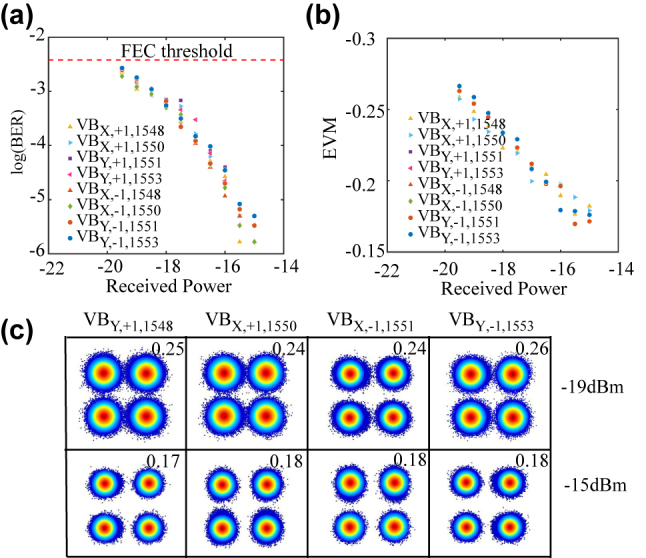
Results for multiplexing communication. (a) BER curve and (b) EVM curve. FEC, forward error correction. (c) Constellations of different channels for different optical power. The EVMs data of Constellations are marked in the upper right corner.

We demonstrate a multiplexing communication (a free-space optical communication link) to further confirm the (de)multiplexing performance of the proposed metasurface. Four *x*-polarization Gaussian beams carrying 25 Gbit/s QPSK signals with four wavelengths (1548.51, 1550.12, 1551.72, 1553.33 nm) are incident on the metasurface at the angles of different diffraction orders. These input beams are converted into VBs with different OAM modes (*l* = ±1) and *x*- or *y*-polarization state, and coaxially transmit along with the zeroth diffraction order. After passing through 2 m free-space, the coaxial VBs incident on another identical metasurface to be demultiplexed and converted into Gaussian beams. Here, two OAM modes, two polarization states and four wavelengths are constructed a 16-channels multiplexing system and the communication capacity achieve 400 Gbit/s (16 × 25 Gbit/s). More details can be found in the Supplementary material. The performance of the communication system is shown in Figure 4. Several BERs and error-vector magnitudes (EVMs) of sixteen channels (two polarization states, two OAM modes, and four wavelengths) versus received optical powers are shown in Figure 4(a) and (b). Corresponding constellations are revealed in Figure 4(c). As can be seen, the BERs are all below the FEC threshold and reach 1.67 × 10^−6^ at a received optical power of −15 dBm. Meanwhile, the EVMs of the constellations decrease to 0.17. These results indicate that these metasurfaces have high transmission efficiency, low crosstalk, and broadband characteristics in multiplexing communication.

## 4 Discussion

Combining conventional multiplexing dimensions involving polarization and wavelength is an efficient means of scaling up the capacity of OAM mode multiplexing communication. In this work, a polarization-dependent phase-modulation metasurface is proposed to simultaneously (de)multiplex the OAM mode and polarization channels. By exploiting the design flexibility of the Dammann vortex grating, different OAM modes with distinct spatial distribution can be customized. And the uniform energy distributions of Dammann vortex grating can profit the (de)multiplexing efficiency. Utilizing the expanded concept of the PB phase, we reveal that the metasurface can impose independent phase profiles of the Dammann vortex grating on two orthogonal linearly polarized lights, which rewards the metasurface by allowing it to (de)multiplex the OAM mode and polarization. Comparing with tradition system via cascading BS or PBS, the Dammann grating design will not increase theoretical loss but effectively reduce system redundancy. Owing to the dispersion-free characteristic of the PB phase, the (de)multiplexer possesses characteristics of a broadband operating wavelength (ranging from 1535 nm to 1607 nm), which enables the (de)multiplexer to be more easily integrated with WDM.

**Figure 5:**
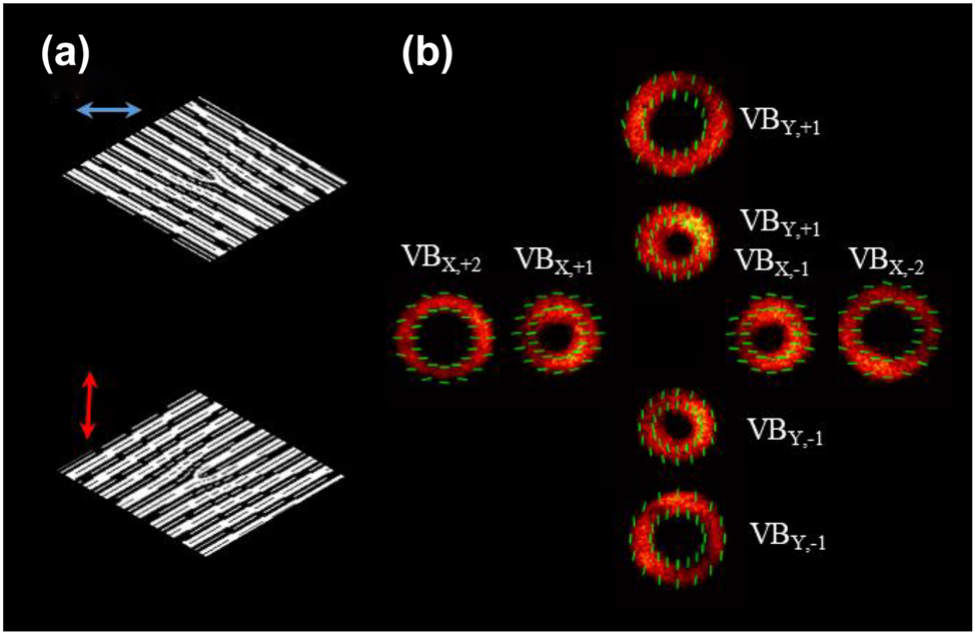
Experiment performance of more OAM modes multiplexing. (a) Two Dammann vortex grating phases with mutually perpendicular off-axis phases. (b) The intensity and polarization distributions of the different diffraction orders.

Communication capacity is a key factor in optical communication and multiplexing more channels is an effective scheme to further enlarge communication capacity. As a proof of concept, 16 channels have been multiplexed, and the communication capacity has reached 400 Gbit/s. To further improve communication capacity, more OAM mode channels can be introduced via the flexible design of the Dammann vortex grating. Here, we further designed and loaded the 1 × 4 Dammann vortex grating phase on the metasurface to generate more OAM modes. To enable different polarization channels to have different spatial channel distributions, the two Dammann vortex gratings have mutually perpendicular off-axis phases, as shown in Figure 5(a). Figure 5(b) illustrates the intensity and polarization distributions of VBs with OAM modes (*l* = ±1, ±2) and *x*- or *y*-polarization states. The beam radius is proportional to the absolute value of the topological charge. These results indicate that it is feasible to multiplex more OAM modes by increasing the diffraction orders Dammann vortex grating. Additionally, the broad working wavelength ranging from 1535 nm to 1607 nm allows it compatible with WDM and multiplexing more than 80 wavelength channels [8, 32].

Finally, the proposed method is also suitable for communication systems with arbitrary beams [41–43]. For example, although cylindrical vector beams (CVBs) have proven to be suitable for long-distance transmission of signals in optical fibers, their demultiplexing remains an unsolved challenge. Considering that CVBs can be regarded as the superposition of VBs with opposite topological charges and the same spatial channel distribution, the Damman vortex grating of our proposed method can be integrated into one metasurface to generate or (de)multiplex radial and angular CVBs. Finally, a complete communication system with CVBs may be achieved.

## 5 Conclusions

In conclusion, we have proposed and implemented a polarization-dependent phase modulation metasurface to simultaneously (de)multiplex OAM mode and polarization channels, and further applied this metasurface to demonstrate OAM mode communication combined with PDM and WDM. We achieved 400 Gbit/s capacity by two OAM modes, two polarizations, and four wavelengths, and the BERs were below 1.67 × 10^−6^ at the received optical power of −15 dBm. Our results may benefit for OAM-based communication, OAM-quantum communication, and large-scale dense data communication.

## Supplementary Material

Supplementary Material Details
