# Supplementary material for: Polarization-dependent phase-modulation metasurface for vortex beam (de)multiplexing
Source: Nanophotonics. 2023 Feb 20;12(6):1129–35. doi: 10.1515/nanoph-2022-0710 (PMC11501251; doi:10.1515/nanoph-2022-0710)
Supplement: Supplementary file 1 — Supplementary Material Details [file j_nanoph-2022-0710_suppl.docx]

Supplementary Material of Polarization-dependent phase-modulation metasurface for vortex beam (de)multiplexing

**Haisheng Wu,^1^ Qingji Zeng,^1^ Xinrou Wang,^1^ Canming Li, ^1^ Zebin Huang,^1^ Zhiqiang Xie,^1^ Yanliang He,^1^ Junmin Liu,^2^ Huapeng Ye,^3^ Yu Chen, ^1^ Ying Li,^1^ Dianyuan Fan,^1^ Shuqing Chen^1, a)^**

*^1^ International Collaborative Laboratory of 2D Materials for Optoelectronics Science and Technology, Institute of Microscale Optoelectronics, Shenzhen University, Shenzhen 518060, China*

*^2^ College of New Materials and New Energies, Shenzhen Technology University, Shenzhen 518118, China*

*^3^ Guangdong Provincial Key Laboratory of Optical Information Materials and Technology and Institute of Electronic Paper Displays, South China Academy of Advanced Optoelectronics, South China Normal University, Guangzhou 510006, China*

^a)^ Author to whom correspondence should be addressed: shuqingchen@szu.edu.cn

**S1.** **The design of individual metaatoms**

The geometric parameters of metaatom are optimum by finite-difference time-domain (FDTD, Lumerical Solutions) based on the finite element method [1]. The heights of Au reflector and SiO2 dielectric spacer are set as *h_1_*=200 *nm*, *h_2_*=150 *nm* to make sure the reflection efficiency. The period of the lattice is chosen to satisfy the subwavelength condition and reduce secondary diffraction. The incident light is set as plane wave with $\lambda$=1550 *nm* and ${45}^{\circ}$ linearly polarized state. After some initial exploration, the height of metaatoms is set as *h_3_*=50 *nm* to acquire large reflection efficiency. The length (*L*) and width (*W*) are swept from 100 *nm* to 700 *nm* to find structure that satisfies half-wave delay. Figure S1(b) and S1(c) the propagation phases as the function of *L* and *W* for x- and y-polarized beam incident. Then several structures satisfied half-wave delay are selected preliminarily. Finally, the structure with the highest reflectivity will be selected for further simulation. In this paper, a metaatom with *L* =380 *nm* and *W* =220 *nm* is selected as basic unit cells to construct metasurface.


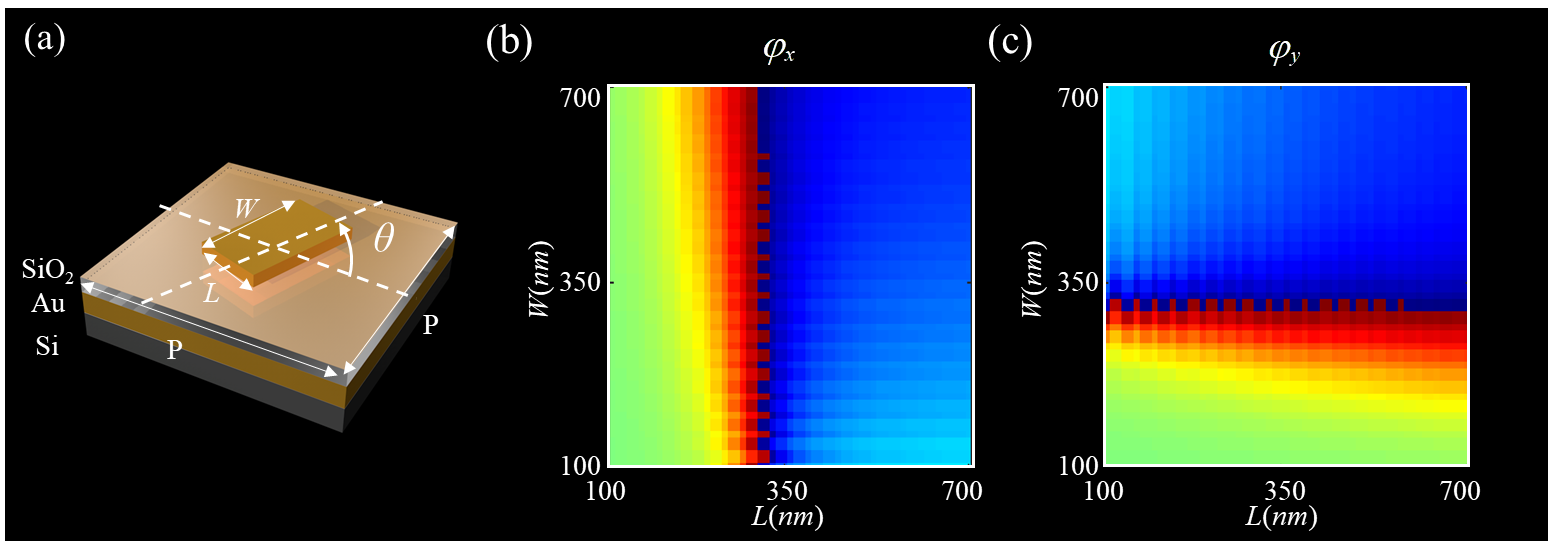


**Figure S1.** The design of individual metaatoms. (a) Structural diagram of metaatom. (b)-(c) the propagation phases of metaatom with different geometric sizes for x- and y-polarized beam incident.

**S2. Simulation of the designed metasurface**


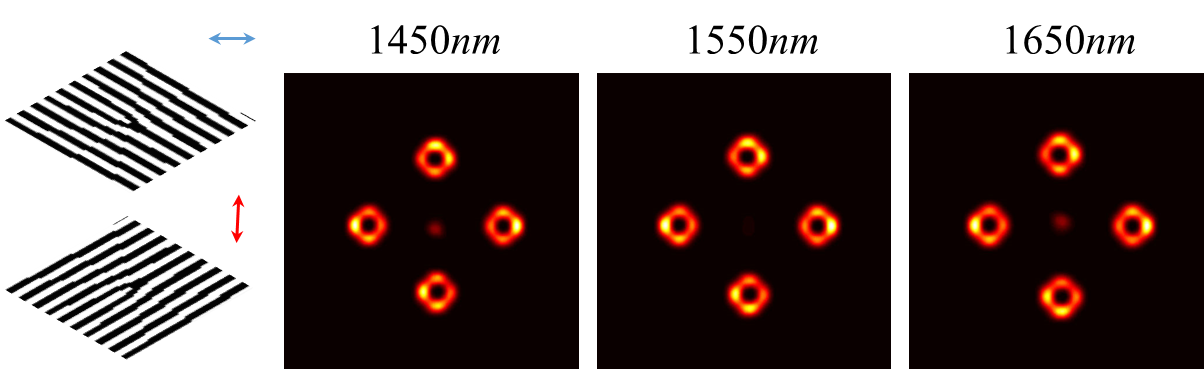


**Figure S2.** Far-field intensity distribution of the metasurface at different wavelengths (1450 *nm*, 1550 *nm*, 1650 *nm*).

In order to verify the feasibility of the proposed metasurface in theory, we simulate the metasurface through the FDTD method. Here, two 1×2 Dammann vortex gratings with orthogonal polarization responses are loaded on the proposed metasurface and illuminated by x-polarized Gaussian beam at different working wavelength (1450 *nm*, 1550 *nm*, 1650 *nm*). As shown in Figure S2, four VBs are generated at the different diffraction orders. The far-field light intensity distributions with different working wavelengths are stable, which verifies the proposed metasurface have a broad working wavelength to compatible with WDM.

**S3.** **Fabrication of the designed metasurface**

We use the standard Electron Beam Lithography (EBL, EBPG 5150) to fabricate the designed metaatoms. Here, 15×15 mm^2^ fused silicon (Si) is selected as substrate and acetone, isopropanol alcohol (IPA), and deionized water (DIW) are used to rinse the substrate in turn before all fabrication process. At the first step, a continuous 200 *nm* Au reflector separated by a 150 *nm* SiO_2_ dielectric spacer is deposited by using the electron beam evaporator (ASB-EPI-C6). In order to enhance the adhesion between layers, 2-5 *nm* titanium (Ti) are used as adhesive. The second step is spin coating the positive polymethyl methacrylate (PMMA, 950K). The spin coating speed and time are set 4000 rpm and 60 seconds to homogenize, then the sample is baked at 180°C for 90 seconds. The thickness of PMMA layer is about 150 *nm*.

Next, the metasurface patterns are written by electron beam lithography (EBPG 5150). The developer and Fixing solution we choose a solution of 3:1 IPA: DIW and DIW. Development and fixation time we set 60 seconds and 30 seconds. Then, the samples are placed in the electron beam evaporator to deposit 50 *nm* of Au film. Finally, the lift-off link we place the sample in acetone solution and let it stand for 5 minutes, then sonicated for 2 minutes to remove the excess PMMA and Au to get the metasurface structure. The above design can ensure a large resonance and high reflectivity at the working wavelength of 1550 *nm*.


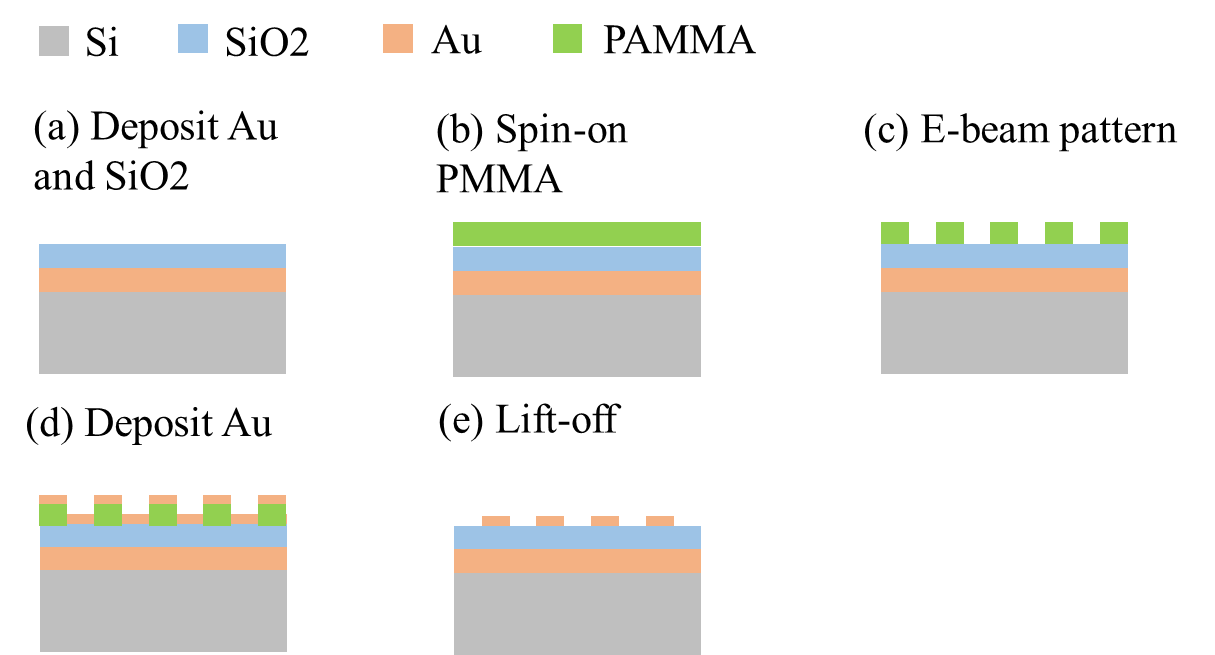


**Figure S3.** Fabrication process. (a) Electron beam deposition of a continuous 200 *nm* Au and 150 *nm* SiO_2_ on a clean fused silica wafer. (b) Spinning of PMMA electron-beam resist layer. (c) Exposure by electron-beam lithography. (d) Electron-beam deposition of 50 *nm* Au. (e) dissolution of PMMA and lifting-off Au.

**S4. Experimental setup of optical characterization**

Figure S4 schematically depicts the setup used for optical characterization test of sample. A 1550 *nm* laser (Thorlabs, HNL210L-EC) provides a laser beam with y-polarization and passes though quarter wavelength plate (QWP) and linearly plate (LP) to generate a laser beam with x-polarization. The overall size of the sample is only 800 × 800 *μm* and the waist radius of incident beam is about 1.5 *mm*, so we use a lens with the focus length *f* =150 *mm* to focus the beam into the center of the sample. To collect and analyze diffraction light field, a beam splitter is placed in the front of the sample and we can collect the reflected light on one side. The observation setup includes a polarization analyzer, and a charge coupled device (CCD) camera (Ophir, SP928). The polarization analyzer, including a QWP and an LP, can analyze the distribution of the polarization state of each diffraction order. And a cylindrical lens (C-lens) can be added to detect topological charges.

**
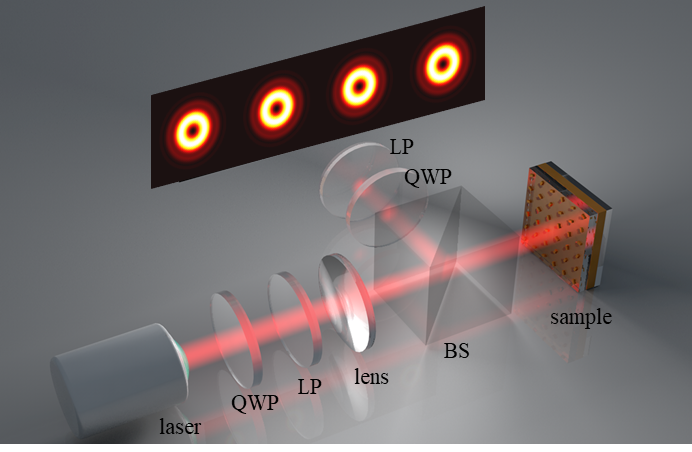
**

**Figure S4.** Optical characterization experimental set-up.

**S5. Broadband property**

To validate the broadband working wavelength of metasurface [2], we captured the intensity and polarization distributions and detect the topological charges at the wavelengths of 1535, 1553, 1589, 1607 *nm* covering from C- to L-bands (Figure S5). The intensity and polarization distributions hardly change with different wavelengths and the diffraction pattern of C-lens demonstrated that the topological charges are not affected by wavelength. From these figures, one can see that metasurfaces possess a broadband working wavelength ranging from 1535 *nm* to 1607 *nm*.


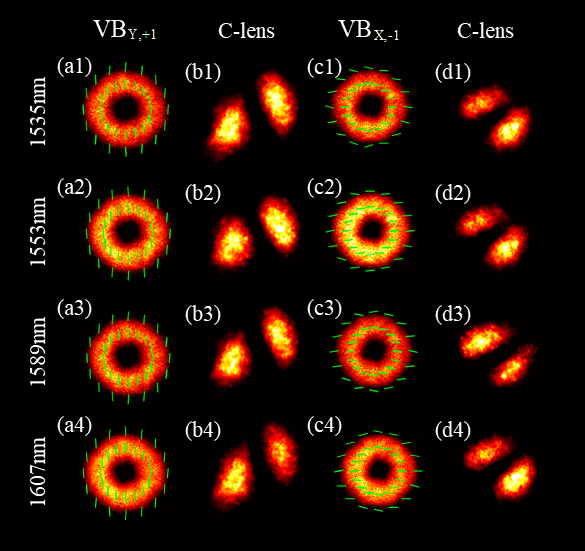


**Figure S5.** Intensity and polarization distributions, and diffraction splitting of the -1*st* and 2*nd* diffraction orders at the wavelengths of 1535, 1553, 1589, 1607 *nm*. (a1)-(a4),(b1)-(b4) The intensity and polarization distributions and the C-Lens detection of the 2nd diffraction orders. (c1)-(c4),(d1)-(d4) The intensity and polarization distributions and the C-Lens detection of the +1st diffraction orders.


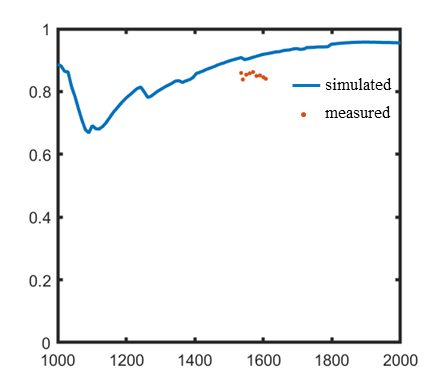


Figure S6. The simulated and measured reflection efficiency of the metasurface

Then, we further measure the reflection efficiency of the proposed metasurface. Gaussian beam is used to normally incident on the metasurface and then an optical power meter is used to record the energy of each diffraction order. The reflection efficiency of the MIM structure is equal to the radio of the total energy of all diffraction orders to the input energy. The simulation result and the measure result are shown in Figure S6. The scatter plots of Figure S6 are the measured reflection efficiencies from 1535 *nm* to 1607 *nm* and all reflection efficiencies exceed 80%. The difference between theoretical and experimental results mainly arise from the experimental errors, including the metasurface fabrication and optical characterization.

**S6. Polarization, wavelength, and OAM-Multiplexing Communication System**

A polarization and OAM-multiplexing communication system [3, 4] combined with WDM is built up to show the (de)multiplexing capacity of the proposed metasurface, as described in Figure S6. The communication system can be divided into three parts: transmitter, free-space optical (FSO) part, and receiver. In the transmitter, four wavelengths, including 1548.51, 1550.12, 1551.72, 1553.33 *nm*, are modulated by IQ modulator and carried 25 Gbit/s quadrature-phase shift-keying (QPSK) signals for WDM. Then the signal light is divided into four subchannels by three optical couplers (OC) and erbium-doped fiber amplifiers (EDFA) are used to amplified signals. At the free-space optical (FSO) part, two identical metasurfaces are used for polarization and OAM mode (de)multiplexing. Four signal beams pass linearly polarizer and incident on the metasurface at the angles of different diffraction orders to generate coaxial VBs with different polarization states and OAM modes (x- and y-polarization and $l=\pm1$). In this work, the diffraction angles are set as about ±5° and ±10° and we need to strictly control the incident angles. Here, two OAM modes, two polarization states and four wavelengths are constructed a 16-channels multiplexing system and the communication capacity achieve 400 Gbit/s (16×25 Gbit/s). After passing through 2 meters free-space, the coaxial VBs incidents on another identical metasurface to demultiplexed and converted into gausses beam with x-polarization. On the receiver end, the QPSK signals are received by an integrated coherent receiver (FUJITSU 100G DP-QPSK Receiver). Finally, we use digital signal analyze (DSA) to further recovery signals, which mainly involves four flow modules: orthogonalization and normalization, clock recovery, frequency offset estimation and carrier phase recovery.

**
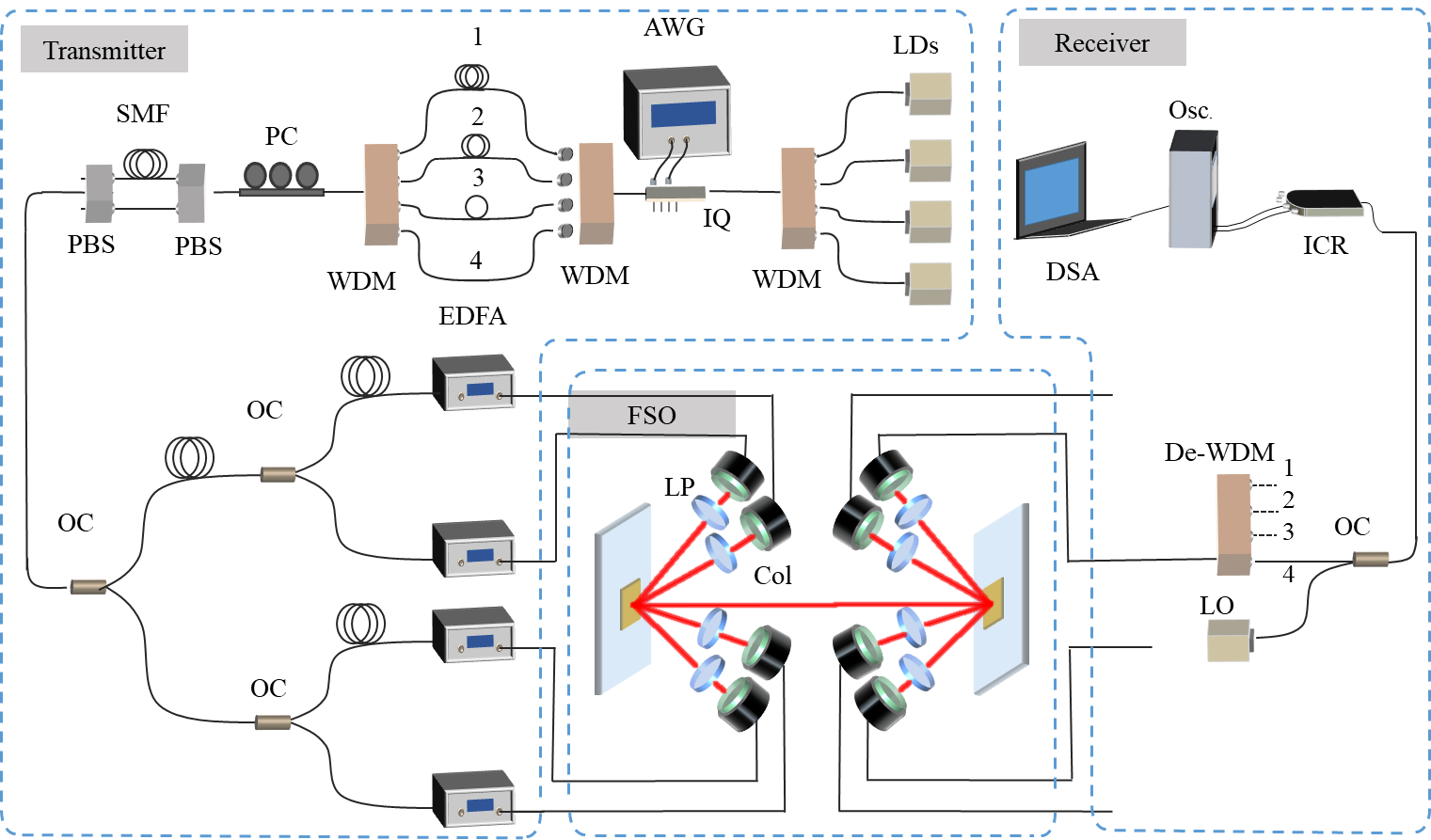
**

**Figure S7.** The multiplexing communication experimental set-up. LD: laser diode; WDM: wavelength division multiplexing module device; IQ: IQ intensity modulator; AWG: arbitrary waveform generator; PC: polarization controller; OC: optical coupler; SMF: single mode fiber; EDFA: Erbium-doped fiber amplifier; LP: linearly polarizer; Col: collimator; ICR: integrated coherent receiver; LO: local oscillator; DSA: digital signal analyze.

**REFERENCES**

[1] B. Yang, Y. He, Z. Xie, J. Liu, H. Ye, J. Xiao, Y. Li, D. Fan, S. Chen, Dielectric metasurface based polarization and orbital angular momentum demultiplexer, Results in Physics, 20 (2021) 103706.

[2] G.-Y. Lee, G. Yoon, S.-Y. Lee, H. Yun, J. Cho, K. Lee, H. Kim, J. Rho, B. Lee, Complete amplitude and phase control of light using broadband holographic metasurfaces, Nanoscale, 10 (2018) 4237-4245.

[3] T. Lei, M. Zhang, Y. Li, P. Jia, G.N. Liu, X. Xu, Z. Li, C. Min, J. Lin, C. Yu, Massive individual orbital angular momentum channels for multiplexing enabled by Dammann gratings, Light: Science & Applications, 4 (2015) e257-e257.

[4] J. Wang, S. Li, M. Luo, J. Liu, L. Zhu, C. Li, D. Xie, Q. Yang, S. Yu, J. Sun, N-dimentional multiplexing link with 1.036-Pbit/s transmission capacity and 112.6-bit/s/Hz spectral efficiency using OFDM-8QAM signals over 368 WDM pol-muxed 26 OAM modes, 2014 The European Conference on Optical Communication (ECOC), IEEE2014, pp. 1-3.
